# Can Traditional Food Product Communication Convey Safety to the Younger Generations? The Role of Sustainable Packaging

**DOI:** 10.3390/foods12142754

**Published:** 2023-07-20

**Authors:** Catia Pasta, Vincenzo Russo, Marco Bilucaglia, Giuseppe Licitra, Guido Mangione, Valeria Micheletto, Federica Rossi, Margherita Zito

**Affiliations:** 1 Consorzio per La Ricerca Nel Settore Della Filiera Lattiero-Casearia e dell’Agroalimentare (CoRFiLaC), 97100 Ragusa, Italy; pasta@corfilac.it; 2 Department of Business, Law, Economics and Consumer Behaviour “Carlo A. Ricciardi”, Università IULM, 20143 Milan, Italy; vincenzo.russo@iulm.it (V.R.); marco.bilucaglia@studenti.iulm.it (M.B.); margherita.zito@iulm.it (M.Z.); 3 Behavior and Brain Lab IULM—Neuromarketing Research Center, Università IULM, 20143 Milan, Italy; federica.rossi31@studenti.iulm.it; 4 Department of Agricolture, Food and Environment (Di3A), Università di Catania, 95123 Catania, Italy; glicitra@unict.it (G.L.); guido.mangione@phd.unict.it (G.M.)

**Keywords:** traditional food product, packaging, food safety, young consumers, dairy, traditional food consumer, sustainability, territory, healthiness

## Abstract

Traditional food products (TFPs) represent a defining part of one’s culture, identity, and heritage with crucial economic, cultural, and environmental benefits in society. Younger generations have a positive idea of TFPs, even if this does not lead to actual purchase, possibly due to the fact that they are often perceived to not meet safety criteria. This study focuses on product communication (CP) and packaging referring to the territory (PT) and to sustainability (SP) in order to verify if these have a direct or mediated impact on perceived product safety (PPS). A structural equation model was conducted on a sample of 1079 young Italian cheese consumers. The results allowed us to confirm the hypothesized impact of CP on PPS through the mediation of PT and, particularly, SP. SP has a crucial communicative role in the model, demonstrating the ability to enhance the perception of the safety of TFPs. This research adds to the knowledge in the field of TFPs, focusing on communication and sustainable packaging as crucial factors conveying healthiness, nutritiousness, and perceived safety, consequently leading to a greater diffusion of the products themselves in the market.

## 1. Introduction

In recent times, despite ever-growing globalization, much attention has been paid to local food production in an effort to enhance social and environmental sustainability [1]. The development of traditional food products (TFPs) has been proven to have many benefits, from the lower impact of short food supply chains with respect to long ones [2,3,4,5] to the protection of existing local economies, along with the promotion of economic growth, enhancement of food safety, activation of social capital, preservation of some areas from depopulation, communication of the territory’s culture, and strengthening of the relationship between consumers and producers [6,7,8].

In line with this, since 1992, the EU has established and implemented a protective agenda for products with locality characteristics, introducing the labels of Protected Designation of Origin (PDO) and Protected Geographical Indication (PGI), together with others [9], which, in Italy, safeguard about 900 products (599 PDOs, 280 PGIs; https://ec.europa.eu/info/food-farming-fisheries/food-safety-and-quality/certification/quality-labels/geographical-indications-register/ [accessed on 3 April 2023]). These forms of promotion and protection also implement food hygiene and safety requirements [10].

Though the label of traditional food product (TFP) has often been reported to be broad, complex, and evolving as a concept [11], it can be defined, in general terms, as the kind of product that is linked to a specific territory, is part of a specific set of traditions, and has shown continuity in its production over time [12]. From a consumer’s perspective, TFPs have been recently defined as “the products frequently consumed or associated to specific celebrations and/or seasons, transmitted from one generation to another, made in specific way according to gastronomic heritage, naturally processed, and distinguished and known because of its sensory properties and associated to a certain local area, region or country” [6]. 

There are valid motives for choosing a TFP across different European countries [13], though Southern Europe is a greater consumer, evidencing a relation between TFP consumption and ethnicity [14]. In general, however, familiarity, a positive attitude toward traditional foods, and the importance of food naturalness and authenticity, together with healthiness, are positively associated with TFP choice [13,15]. Furthermore, Fernandez-Ferrin et al. [16] categorized choice motives into two types of factors: on the one side, paying attention to rural communities and environmental benefits, considered as public factors, and, on the other side, the search for nutritional benefits or food safety, considered as private factors.

Indeed, TFPs carry a strong symbolic value [6], and there is also an emotional association linked to words such as natural, traditional, homemade, and authentic, the intensity of which actually favor TFP choice [13]. Additionally, experimental studies based on neuromarketing techniques even confirmed that emotions are a crucial factor in designing an effective communication campaign for TFPs [17]. 

What emerges from Pieniak’s study [13] is that, in contrast with what was mentioned above, a negative relation between TFP and healthiness comes to light, confirmed also by Caputo et al. [15], who affirmed that healthiness can be either positively associated with TFP choice or a limiting factor inhibiting the consumption of TFPs. This means that traditional foods are not unanimously perceived as healthy, safe, and nutritious, as may have been imagined. The reason that is suggested is that perhaps they are often perceived as too high in fat, cholesterol, and/or sugar; as too energy-dense; and, due to minimal conservation processes, as presenting higher microbial risks. This was also confirmed by Guerrero et al. [6].

According to Vanhonacker et al. [14], TFP consumption is not even across Europe. The typical traditional food consumer is someone who lives in Southern Europe, is over 40, considers him/herself health-conscious, spends time cooking, spends a good deal of money on food, looks for familiarity in food, and has a very positive attitude towards traditional food products. On the other hand, the typical non-traditional food consumer is young, weight-conscious, open to technology and innovation in food, and seeks value for money [14]. Other studies confirm that young people actually have a very positive idea of TFPs, which they consider good [9], very satisfactory, and of significant quality [16]. They often consider them as natural [16]. However, particularly young southern Europeans feel that they do not trust these products to be safe; this is negatively correlated with age [9]. 

Indeed, safety is a key factor and can be intended in a broad or a narrow sense [18]. In a narrow sense, food safety is conceived as the opposite of food risk and in a broad sense, it can be viewed as encompassing the nutritional health qualities of food. In the present paper, we refer to product safety in the broad sense, meaning the perceptions a consumer has about the health and nutrition of a product [18,19]. 

In the literature, concern for food safety and health consciousness are among the major factors that play a crucial role in the formation of attitudes towards food [20]. Indeed, safety has been proven to have an impact on TFP choice [13,21], on the willingness to pay, in the case of high-quality food [18], and on the intention to buy [19]. Thus, TFPs should feel safe to consumers in terms of their ingredients, production process, and packaging so as to induce positive attitudes and not to hinder product choice and then purchase. Cerjak et al. [22] emphasize the fact that communication is a crucial activity to make the consumer aware about the naturalness of a product, the use of traditional ingredients, the intrinsic quality criteria for the choice of raw material, and other information concerning the origin and the history of a product.

Indeed, product communication is crucial for the knowledge and promotion of a TFP and its associated territory, starting from its origin, its history, the first method of production or development, and the history and evolution of the producers and the place in which it was created. Furthermore, knowledge of the site of production of a product has a significant and positive impact on consumer preferences [23]. These factors increase product knowledge and reinforce product experience [24] and are considered important drivers for TFP choice [6,14,22]. Packaging itself can have an important role in TFP communication. 

Today, packaging is no longer a mere structural element for food storage and preservation, but also a powerful marketing tool that can affect product perception [25,26], purchase decision, and food choices [27,28,29,30]. According to van Rompay and Veltkamp [31], food packaging might represent the most direct and influential communication element at the point of purchase. More than half of newly launched products fail [32], and in some cases, this is a result of poor packaging design that does not meet consumers’ demands and expectations [33]. Therefore, understanding consumers’ responses to food product packaging seems to be relevant for maximizing package impact [33] and it seems to be even more important for local traditional foods.

On the one hand, packaging can be the expression of a territory and communicate information about the origin of the product and its tradition, thus conveying ideas of authenticity and naturalness. All background information about the product origin is important for the consumer’s evaluations of product quality, product value, and willingness to buy, and is related to product purchase [34,35]. Finally, the reference to the territory benefits both the brand and the product [36]. 

On the other hand, packaging can focus on sustainability. Today, consumers are well informed about the impact of packaging on the environment and therefore increasingly look for packaging made from recycled materials that produce as little waste as possible when finished [37] such as glass and cardboard, which are perceived to be more environmentally friendly. According to a study by the Nomisma Consumer Packaging Observatory [38], one in four Italians indicate the presence of sustainable packaging as their main driver of food choice. Moreover, 14% claim to have stopped buying some products because the packaging did not contain sustainable elements. Nevertheless, consumers are much more likely to prefer environmentally friendly products [39,40], especially if the aesthetic design is of high quality [41,42], and young consumers in particular are more environmentally aware and more oriented towards sustainable packaging [43].

Furthermore, considering the importance of a more widespread presence of TFPs in the market, while many studies have focused their attention on the drivers for TFP choice [9,13,15], a few studies have analyzed the concept of food safety in relation to TFPs [17,19], and further consideration still needs to be given to the association between food safety and the concept of sustainability conveyed through packaging. Moreover, considering that younger generations will be the consumers of the future, playing a future key role in the communication of safe, environmentally friendly, and territorial products, it is important to investigate and monitor this group of people in order to understand how to present these products for consumption and also create environmentally friendly conditions. 

Research on the packaging of traditional food products as a communication tool through which the product origin and history can be communicated and also on the possible relationship it can have with the consumer’s perception of healthiness and nutritiousness [44] is crucial to the deepening knowledge in this field.

On the basis of the above, the main aim of this study is to investigate young consumers’ perception of product safety as a result of product communication through two different types of packaging of dairy products, either related to the territory or to sustainability attributes and materials. The use of dairy products is due to the fact that they are products that are strongly connected to the territory in their production process, as was also studied by Russo et al. [19]. The subject of the analysis was young Italian consumers. Recent studies show that Italians tend to buy cheeses for family and household consumption, evidencing that for Italians, family tradition and the convivial food sharing of dairy products are absolutely fundamental [45]. Within the dairy sector, it is known that nowadays, consumers are more aware of aspects such as health, quality, sustainability, and territory [46]. According to the FAO’s 2022 report, cheese is the second most important product in the dairy sector after milk, with an estimated export volume of about 3.5 million tonnes per year worldwide [47]. This justifies our choice of cheese as a representative object of study for communication in the dairy sector.

We are hereby considering that packaging can be an important means of communication, that food packaging plays an important role in general knowledge of the product, and that the communication of the history and origin of the product can be coherently conveyed through the packaging. By using the right aesthetics, materials, and information, the focus can be either on the relation with the territory [19] or on the attention to the environment and sustainability [44,48]. Thus, we can hypothesize the following:

**H1a.**  *Communication about the story and the origin of the product (CP) has a direct positive association with the type of packaging linked both to the territory (PT) and sustainability (SP).*

Furthermore, while certifications or country-of-origin claims play a role and can build solid product reputation and increase perceived product safety and healthiness [49], the perception of product safety, which is subjective and resides in the mind of the consumer [18], can be influenced by proper communication about the detailed history and origin of both product and producer, or indications about the place of production, which can be useful in increasing knowledge and trust of consumers in the product and its reliability [50]. Thus, the second hypothesis is as follows:

**H1b.**  *CP has a direct and positive association with the perception of safety of the product (PPS).*

Packaging should protect food and be safe in order to keep TFPs healthy, nutritious, and of high quality [44] but it should also be able to convey reassuring messages about the safety of the product [19]. According to Bou Mitri et al. [44], the characteristics of the packaging—in terms of shape, text, images, and material—can change the perception of safety. For instance, in the case of cheese, vacuum packaging is the most appreciated because it is perceived as the highest quality and healthiest packaging [44]. Indeed, transparent color is perceived as an important feature conveying safety, especially for fresh food [25,44]. Finally, the presence of nutrition and health claims, ingredients, and allergens (if any) on the packaging is also related to safety [44]. On the other hand, packaging with natural color and natural materials conveys an impression of authenticity, healthiness, and quality [51].

In the literature, an association has been found between packaging referring to the territory and perceived product safety [19], but also between environmentally friendly and sustainable packaging and the food quality and safety [52,53]. Therefore, we hypothesize the following:

**H2.**  *Both the type of packaging PT and SP have a direct and positive association with PPS*.

Finally, food packaging plays an important role in not only attracting consumers’ attention but also conveying meaning and generating expectations [26]. In this study, we considered packaging that evokes the territory and conveys its tradition, authenticity, and naturalness, and sustainable packaging that conveys concepts like environmental awareness, authenticity, and responsibility. We hypothesized that on the basis of what was mentioned above, the packaging could be a mediator that reinforces the communication of TFPs as products linked to the territory, their origin, and their history, so that the consumers increase their perception of food healthiness and safety [44]. Thus, we can hypothesize the following:

**H3.**  *The association between CP and PPS is mediated by PT and SP*.

In Table 1, all abbreviations for the measures of the studied variables are given. 

## 2. Materials and Methods

### 2.1. Participants and Procedure

A self-report questionnaire was administered on an online platform in Ragusa, Italy (Google Modules), to a sample of young Italian consumers (N = 1166) between 18 and 35 years old. Before completing the questionnaire, all participants gave their informed consent directly online in a dedicated field, along with privacy information. The questionnaire included a cover letter to which researchers attached instructions and information about voluntary participation in the study and anonymity. The study complied with the Declaration of Helsinki (World Medical Association [54]) and the General Data Protection Regulation. Ethical approval was not necessary because the study did not provide medical treatments or other practices that can be the cause of psychological or social problems to participants. The questionnaire was administered between December 2021 and April 2022. Before completing the questionnaire, participants were asked to provide demographic information and self-report on screening questions related to cheese consumption (i.e., whether they purchase and eat cheese), diet (i.e., whether they follow a specific diet and if so, which diet), and cheese allergies (i.e., whether they are allergic or intolerant to lactose and dairy products). Overall, this information provided an indication of whether or not respondents consumed cheese on a regular basis. Participants who did not meet the criteria concerning age and actual cheese consumption were not included in the study.

After the data cleaning according to the above criteria, the final sample consisted of 1079 young Italian cheese consumers (59% females), with a mean age of 25 years (SD = 3.84, range 18–35). Most of them were students (50.6%) or workers (46%) and only 3.4% were unemployed. Less than half of the sample (45.5%) had a university degree (29.7% undergraduate and 15.8% postgraduate). Among them, 63.9% lived with their family of origin, 10.8% lived with friends/roommates, 10.5% lived with their partner, 8% lived alone, and 6.7% with their partner and children or just with their children. The average number of children was 0.14 (SD = 0.51, range 0–4). 

The number of daily meals distributed in the sample was as follows: 48% from 1 to 3, 50% from 4 to 5, and 2% over 5. While only 9.2% of the participants considered themselves “cheese expert”, 62.6% of them knew the difference between certified (i.e., PDO/PGI/TSG) and non-certified cheese, and 96.9% of them trusted the former. Regarding weekly consumption frequency, the “type of cheese” factor (3 levels: fresh, semi-matured, and matured), investigated through 3 Likert scales (4 points: 1 = never, 2 = 1 or 2 times, 3 = 3 or 4 times, 4 = 5 or more times) was found not to be significant according to the Friedman test (χ^2^(2) = 2.269, *p* = 0.322). 

Regarding the frequency of cheese purchase, the factor “buyer” (5 levels: self, parents, relatives, friends, and others), investigated through 5 Likert scales (7 points, 1 = never, 7 = always), was found to be significant, as confirmed by the Friedman test (χ^2^(4) = 1996.744, *p* < 0.001). Conover’s post hoc comparisons with Holm’s correction showed that cheese was most frequently purchased by parents (M = 4.209, SD = 2.076), followed by themselves (M = 3.062, SD = 0.057), relatives (M = 1.872, SD = 1.501), friends (M = 1.511, SD = 1.220), and others (M = 1.387, SD = 1.160). 

### 2.2. Measures

In accordance with the above literature and the aim of this study, this research used four main measures to assess young consumers’ attitude towards dairy products (the list of items is shown in Table 1). All measures required participants to indicate their agreement or disagreement with each item on a 7-point Likert scale ranging from 1 (strongly disagree) to 7 (strongly agree).

The first measure is communication about the story and origin of the p (CP): based on the assumption about the role of communication in explaining the history of the product and the territory where it is produced [8,19], the following three items were formulated: “it is important to know the story of the product”; “it is important to know the story of producers”; “it is important to know the places of production”. 

The second measure is packaging and territoriality (PT): we used three items that were already formulated and successfully administered in a previous study by Russo et al. [19]. In this study, we considered this type of packaging as more linked to process and traditions. The items are “the packaging must reflect the tradition of production”; “the packaging must recall the territory”; “the packaging must clarify the place of production”. 

The third measure is sustainable packaging (SP): based on the considerations about the characteristics of sustainable food packaging [25,44,55], we formulated the following three items: “the packaging must be reusable”; “the packaging must be compostable”; “the packaging must be biodegradable”. 

The fourth and outcome measure is the perceived product safety (PPS): this measure, previously used in a study by Russo et al. [19], focuses on consumers’ perceptions of how healthy and nutritious a product is. The items are as follows: “it is important that the products I choose are healthy”; “it is important that the products I choose are nutritious”; “it is important that the products I choose are without additives”.

### 2.3. Data Analyses

In the data analyses, descriptive statistics, correlations (Pearson’s r), and alpha reliabilities (α) for each scale were calculated using SPSS 27. 

Twelve linear regressions were fitted through JASP to predict the item scores (CP1-3, PT1-3, SP1-3, PPS1-3) from the socio-economic variables to investigate their possible contribution to the subsequent structural equations model (SEM). Gender (2 levels: male and female), living status (5 levels: living alone, living with their family of origin, living with friends or roommates, living with a partner, living with a partner and children or with children), educational level (3 levels: no university degree, undergraduate, postgraduate), and occupation (3 levels: student, worker, unemployed) were considered as factors, while age and number of children served as covariates. The adjusted R^2^ and the root mean square error (RMSE) were calculated as goodness of fit, while the significance of the coefficients was assessed using ANOVA (df_num_ = 11; df_den_ = 1019). The family-wise error rate associated with the regression fits was controlled using the Bonferroni method. 

To estimate SEM, MPLUS 8 was used to test the relationship between the detected variable and the mediation of the two types of packaging (PT and SP, suggesting different information about the product) from the communication of product (CP) to perception of safety (PPS). In this study, the hypotheses were specified a priori and a partial mediation model was used [56]. The goodness of fit of the model was assessed using the chi-square value (χ^2^), the comparative fit index (CFI), the Tucker–Lewis index (TLI), the root mean square error of approximation (RMSEA), and the standardized root mean square residual (SRMR).

The significance level for all statistical analyses was set at 0.01.

To assess the possible effects of common method bias, Harman’s single-factor test was performed [57] through a confirmatory factor analysis. The results obtained with MPLUS 8 showed the following fit indices: χ^2^(135) = 5478.344, *p* < 0.00, CFI = 0.63, TLI = 0.58, RMSEA = 0.19, SRMR = 0.10. These indices show that the model cannot be identified, thus indicating that one single factor is not accounting for the variance in the data and suggesting that the common method is unlikely.

## 3. Results

The psychometric analyses showed satisfactory Cronbach’s alphas for all the assessed variables, ranging between 0.80 and 0.90, thus meeting the criterion of exceeding 0.70 [58]. 

Moreover, to test the validity of each scale, confirmatory factor analyses were performed with MPLUS 8, which showed very good results, with factor loading exceeding the value of |0.30|, indicating a good correlation between the factor and the item [59]. In particular, the analyses suggested the following: 

CP obtained a very satisfactory reliability with a Cronbach’s alpha coefficient (α) of 0.88. The confirmatory factor analysis revealed a single-factor model with satisfactory fit indices: χ^2^(3) = 2129.896, *p* < 0.00, CFI = 1, TLI = 1, RMSEA = 0, SRMR = 0. The factor loadings were also satisfactory and were, respectively, |0.95|, |0.92|, |0.65|.

PT obtained a satisfactory reliability with a Cronbach’s alpha coefficient (α) of 0.83.

Confirmatory factor analysis resulted in a single-factor model with satisfactory fit indices: χ^2^(3) = 1234.426, *p* < 0.00, CFI = 1, TLI = 1, RMSEA = 0, SRMR = 0. Factor loadings were satisfactory and as follows: |0.72|, |0.81|, |0.83|.

SP obtained a good reliability with a Cronbach’s alpha coefficient (α) of 0.80. Confirmatory factor analysis showed a single-factor model with satisfactory fit indices: χ*^2^*(3) = 1424.957, *p* < 0.00, CFI = 1, TLI = 1, RMSEA = 0, SRMR = 0. Factor loadings were satisfactory and, respectively, |0.72|, |0.96|, |0.73|.

Finally, PPS showed a very satisfactory reliability with a Cronbach’s alpha coefficient (α) of 0.90. Confirmatory factor analysis showed a-single factor model with satisfactory fit indices: χ*^2^*(3) = 2038.686, *p* < 0.00, CFI = 1, TLI = 1, RMSEA = 0, SRMR = 0. Factor loadings were satisfactory and as follows: |0.82|, |0.92|, |0.86|.

None of the regression models were found to be significant after correcting the significance level at 0.01/14 = 0.0008, suggesting that the socio-economic variables had no effect on the items. The results are shown in Table 2. 

The correlations are shown in Table 3 with descriptive statistics of the detected measures. With regard to descriptive statistics, each variable exceeds the central point of the scale, with higher levels of PPS and CP in this sample. With regard to the correlations, this sample shows high, positive, and significant correlations within all the assessed variables, with higher correlations between PT and SP (*r* = 0.73), between CP and PT (*r* = 0.61), and between CP and SP (*r* = 0.55). In addition, PPS has positive and quite high correlations with the other variables, especially with the two types of packaging (*r* = 0.48). 

**Figure 1:**
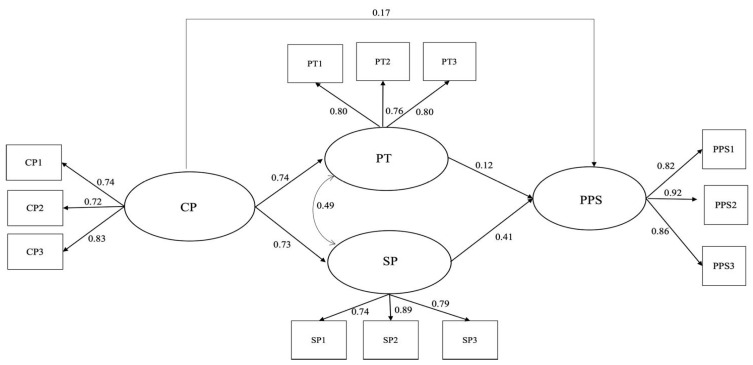
Results of the multi-group structural equations model. Note. CP = communication about the story and origin of the product; PT = packaging and territoriality; SP = sustainable packaging; PPS = perceived product safety.

The estimated SEM (Figure 1) showed satisfactory fit indices, confirming the goodness of model fit: χ^2^(45) = 331.744, *p* < 0.00, CFI = 0.97, TLI = 0.95, RMSEA = 0.07; SRMR = 0.03. Moreover, the SEM showed significant and good item loadings (*p* < 0.001), suggesting a good structure of the latent variables. In this model, CP showed a direct, positive, and strong association with both PT (*β* = 0.74) and SP (*β* = 0.73) and a lower but significant and positive association with PPS (*β* = 0.17). The two types of packaging show different associations with PPS; PT, in fact, shows a lower positive and significant association with PPS (*β* = 0.12) than the association between SP and PPS (*β* = 0.41).

Moreover, the model detected the mediating role of PT and SP between CP and PPS. As shown in Table 4, two significant mediations were found among the two mediators, with a higher level in the case of SP as a mediator (*β* = 0.30) than PT (*β* = 0.10).

## 4. Discussion and Conclusions

This study is based on the idea that a greater market presence of traditional foods could have important implications for growth opportunities in the territory they come from [24]. In order to increase the market presence of TFPs, we hypothesized that the communication of the product, in a direct way or through the mediation of different types of packaging—either with a stronger link to the territory and tradition or with a stronger link to sustainability—could have a positive association with the perceived safety, which has been recognized in the past as having a positive relation with product choice, willingness to pay, or purchase intention [13,18,19,21]. For this reason, we investigated consumers’ disposition towards traditional dairy products based on four main variables: communication about the story and origin of the product (CP), packaging and territoriality (PT), sustainable packaging (SP), and perceived product safety (PPS). Our results highlight that this type of products, such as those offered in sustainable packaging, can convey a perception of safety, which would then impact the product choice. 

Younger generations tend to anticipate future consumption trends and also show a greater responsiveness to change [60]. Thus, they were selected as the target group. This selection was also due to the combination of two further reasons. On the one hand, younger generations are not so keen to consume TFPs for many reasons, among which safety is crucial [9]. On the other hand, even though sometimes younger generations show a contradiction between what they know and appreciate about sustainability and what they actually practice [61], they are generally considered socially, economically, and environmentally conscious and discerning [62]. 

We believe that this study contributes to the literature because it sheds new light on perceived food safety as a driver of TFP choice and sustainability as a component of TFP communication, confirming the importance of sustainable packaging in communicating the nutritional value and healthfulness of the product, especially to younger generations. This paper can be an interesting guide to develop the right marketing strategies to spread TFPs among young people.

Our first results come from the descriptive statistics, from which we can see how much importance consumers attribute, first, to the perception of safety intended as healthfulness and nutritiousness of the product and, second, to the knowledge of the history of the product and the producer of traditional dairy products. Here, it is also possible to see that there is a difference between the two kinds of packaging and that the sustainable one seems to be more important to the consumers than the traditional one.

Furthermore, the correlations between all variables are significant and moderately strong, proving the relationship between all the variables. Indeed, changes in packaging or in product communication are positively related to changes in perceived safety. It is also interesting to note that a strong correlation exists between sustainable packaging and the packaging related to the territory, suggesting that there is a connection in the mind of the consumer between these two elements based on a common ground of naturalness, authenticity, and genuineness, which should be further investigated. Moreover, this is in line with studies suggesting a preference for the product and its communication and sustainable packaging among young people [43], suggesting this target is an important key point worthy of attention. 

Considering the hypotheses that we made, the first hypothesis, H1a, assumes that there is an association between the traditional product communication and packaging related to the territory or sustainable packaging. The strong association that emerges from the analysis leads us to confirm the hypothesis and suggest that the packaging is a crucial means to communicate the territory of the product, what the product is, how it is made, and the link between the product identity and its territory. The packaging—either related to the territory or created with environmentally friendly materials and limiting over-packaging and waste [63]—is the visual expression of the story of the product and the manufacturer, which is also in line with Hidayanto et al. [64].

Hypothesis H1b suggested that the communication of the product has a direct impact on the perception of safety of the product, which is confirmed by the data, even though a very strong association is found with the packaging in the next confirmed hypotheses. However, this point clearly indicates that the consumer tends not to infer the safety of the product directly from its history and origin. Indeed, he/she needs to see more than just the origin information of the product in order to have an idea of the healthiness and nutritiousness, which suggests that another lever like packaging might be necessary in order to enhance efficacy. Local production, which is related to aspects of territorial promotion and sustainability, is particularly closely linked to health, which is consistent with a previous study by Russo et al. [19]. Health is also important among younger generations. 

H2 states that both the types of packaging have a direct and positive association with the perception of safety of the product. The aspect of territory is very important, but its effect is not so strong as to reassure the consumers about safety. Sustainable packaging is much more effective, which is in line with the literature that emphasizes that attention to territory can be enhanced through environmentally friendly elements [51]. With regard to sustainable packaging, we know that concern for the environment and reduced risk to health are two main motives for buying food products in environmentally sustainable packaging [65]. This is a crucial result because unlike what is presented in the literature, where safety is mainly associated with the sustainable processing and production areas [66,67,68], in this case, safety is strictly linked to the sustainable packaging. This means that communication elements, such as packaging, add value to the perception of sustainable and healthy products. Moreover, the fact that sustainable packaging is a tangible feature of the product allows the producers to clearly communicate their sustainable actions, building a relationship of trust with the consumers and avoiding the risk of being interpreted as greenwashing. 

Also, among the younger generation, the packaging acts as a mediator for product communication, reinforcing and enhancing the association with PPS and clarifying the necessity for consumers to visualize, in particular sustainable packaging, in order to perceive TFP safety, which is in line with the literature [69], confirming Hp3.

As far as possible applications are concerned, the focus on packaging is crucial as it clearly communicates the link with tradition and territoriality. The local aspect, if it is sustainable, has a much stronger impact on the salubrity of traditional food products. Traditional communication has an impact on how the product is perceived and packaging has a positive association with the perception of product safety, but when you look at mediation, sustainability has a very important effect in the process.

This represents a real challenge for both product marketing and future product development in the traditional food sector, as it provides an indication of how the traditional food consumer could be better reached and how the packaging of these type of products can be crucial for the knowledge of the TFP brand and the choice of TFPs among the young generations.

Although consumers rationally report a preference for sustainable packaging and, in particular, most consumers indicate they want to purchase products that use as little packaging as possible, a study on the value–action gap found that some environmentally friendly packaging may require customers to make tradeoffs between product quality, performance, and price; this creates a different “attitude-behavior” in consumers [39], which leads people not to buy a product due to economic, socio-economic, and demographic factors [70], so the sustainability features of packaging do not always translate into a willingness to purchase [71]. Therefore, we suggest that further studies be conducted on the role of sustainable packaging. This would be useful in both promoting environmental protection and awareness in this area and attracting the interest of young consumers, as well as ensuring the longevity of the territorial products and their manufacturers. In fact, territorial and local products are not only considered safe and natural, but are also associated with emotions and authenticity [6,13], which strengthens affective engagement to products and producers. This is linked to another important challenge when it comes to marketing and government policies: awareness of the importance of territorial products is a key concept to protect territorial productivity and economies. Moreover, promoting sustainable processes and principles is a critical element for the planet and economic development. Through the recognition of the relationship between key variables, the challenge is to raise awareness among the younger generation, as they will be the future adults in the future economy. They will play a key role for the planet and also in terms of a community vision: consuming local products will boost the local economy; consuming sustainable products will protect the planet; conscious consumption will have less impact on the environment and overall public health spending.

Stimulating such considerations and allowing critical reflection to open up in scientific and policy debates is a practical contribution of this study. 

Considering that well-being is such an important issue for human beings [72], it would be interesting to understand whether perceived healthiness and safety can actually have an impact on well-being itself or at least be intertwined with it. 

### Limitations and Future Avenues

Finally, apart from these contributions, it must also be acknowledged that this study has limitations in terms of the sample, which is non-probabilistic and includes only Italian people. This does not allow us to generalize the data, even though they provide the first critical points that should be considered both in research and the actions of governments to support sustainability and territorial policies among such young target groups, future adults and consumers with responsibilities for the fate of the planet. Future research approaches should widen the survey to include consumers living in different areas, involving different age groups to compare the perception and experiences of such issues related to sustainability and provenance, and study informational labels on packaging that can increase perceptions of sustainability to support product safety for traditional cheeses to compare with and improve the outcomes of this study. Moreover, future studies can compare, through a simultaneous analysis with a multi-group structural equations model, the experience of such variables among cheese and non-cheese consumers in order to better capture important differences (or similarities) between consumers. A more in-depth study of this topic should allow firms to not only understand which elements of on-package communications are important to different consumers but also which innovations and/or changes might be introduced for traditional foods in general, based on yet-to-be-discovered common traits. Finally, neuromarketing techniques would be useful to evaluate and assess emotional aspects and activations toward the territorial and sustainable stimulations [17,19], since neuromarketing allows the detection of the emotional part of consumers, overcoming the rationality [73].

## Figures and Tables

**Table 1:** List of measures and items.

Abbreviation	Measure	Items
CP	Communication about the story and origin of the product	1. It is important to know the story of the product 2. It is important to know the story of producers 3. It is important to know the places of production
PT	Packaging and territoriality	1. The packaging must reflect the tradition of production 2. The packaging must recall the territory 3. The packaging must clarify the place of production
SP	Sustainable packaging	1. The packaging must be reusable 2. The packaging must be compostable 3. The packaging must be biodegradable
PPS	Perceived product safety	1. It is important that the products I choose are healthy 2. It is important that the products I choose are nutritious 3. It is important that the products I choose are without additives

**Table 2:** Results of the regression models for each item and each measure with the indices of fitting (adjusted R^2^ and RMSE) and the results of the associated ANOVA (*F*-value, *p*-value).

Measure	Item	Adjusted R^2^	RMSE	*F*	*p*
CP	1	0.005	1.961	1.473	0.136
	2	0.000	1.857	0.963	0.478
	3	0.002	1.997	1.176	0.299
PT	1	0.013	2.022	2.231	0.011
	2	0.003	1.946	1.238	0.257
	3	0.050	1.962	1.486	0.131
SP	1	0.020	2.076	1.878	0.038
	2	0.024	2.024	2.217	0.012
	3	0.029	1.937	2.697	0.002
PPS	1	0.016	1.961	1.473	0.136
	2	0.000	1.857	0.963	0.478
	3	0.013	1.977	1.176	0.299

Note. The reported *p*-values must be compared to the Bonferroni-corrected significance level 0.01/14 = 0.0008. CP = communication about the story and origin of the product; PT = packaging and territoriality; SP = sustainable packaging; PPS = perceived product safety.

**Table 3:** Means (M), standard deviations (SD), and correlations (Pearson’s r).

	M	SD	1	2	3	4
1. CP	4.17	1.68	(0.88)			
2. PT	3.73	1.67	0.61 **	(0.83)		
3. SP	3.83	1.69	0.55 **	0.73 **	(0.80)	
4. PPS	4.92	1.76	0.42 **	0.48 **	0.48 **	(0.90)

Note. ** *p* < 0.01 level. Cronbach’s alphas are on the diagonal (between brackets). CP = communication about the story and origin of the product; PT = packaging and territoriality; SP = sustainable packaging; PPS = perceived product safety.

**Table 4:** Indirect effects of the estimated multi-group SEM.

Indirect Effects	Standardized Indirect Effects		
	Est.	s.e.	*p*
CP → PT → PPS	0.10	0.04	0.00
CP → SP → PPS	0.30	0.04	0.01

Note. CP = communication about the story and origin of the product; PT = packaging and territoriality; SP = sustainable packaging; PPS = perceived product safety.

## Data Availability

The datasets generated for this study are available on request to the corresponding author.
